# Application of 5G network combined with AI robots in personalized nursing in China: A literature review

**DOI:** 10.3389/fpubh.2022.948303

**Published:** 2022-08-24

**Authors:** Caixia Guo, Hong Li

**Affiliations:** ^1^Presidents' Office, China-Japan Union Hospital, Jilin University, Changchun, China; ^2^Department of Emergency Medicine, China-Japan Union Hospital, Jilin University, Changchun, China

**Keywords:** 5G network, personalized nursing, AI, cloud platform, robotics

## Abstract

The medical and healthcare industry is currently developing into digitization. Attributed to the rapid development of advanced technologies such as the 5G network, cloud computing, artificial intelligence (AI), and big data, and their wide applications in the medical industry, the medical model is shifting into an intelligent one. By combining the 5G network with cloud healthcare platforms and AI, nursing robots can effectively improve the overall medical efficacy. Meanwhile, patients can enjoy personalized medical services, the supply and the sharing of medical and healthcare services are promoted, and the digital transformation of the healthcare industry is accelerated. In this paper, the application and practice of 5G network technology in the medical industry are introduced, including telecare, 5G first-aid remote medical service, and remote robot applications. Also, by combining application characteristics of AI and development requirements of smart healthcare, the overall planning, intelligence, and personalization of the 5G network in the medical industry, as well as opportunities and challenges of its application in the field of nursing are discussed. This paper provides references to the development and application of 5G network technology in the field of medical service.

## Introduction

With the continuous progress of information technology, the informatization degree of the medical field is continuously increasing, the business of information systems becomes increasingly complicated, and the size of daily data to be processed in hospitals is rapidly increasing. Meanwhile, the development of regional medical and healthcare demands network interconnection and real-time sharing of medical data. As a result, the communication network of the medical industry is facing huge challenges. In the post-epidemic era, a reform in the medical service mode to provide personalized service is of great significance.

**Figure 1:**
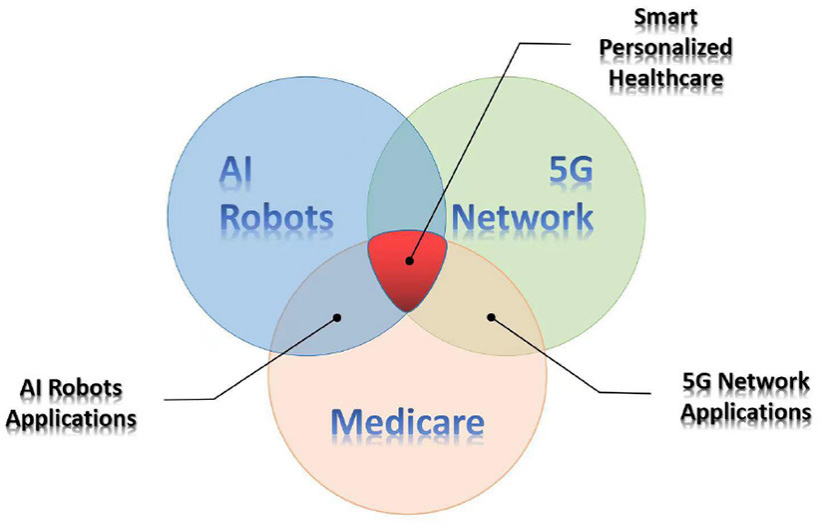
Logical relation diagram.

Intelligent medicine has been applied to all aspects of the medical system, such as health care, medical auxiliary diagnosis, and hospital management. A series of cutting-edge technologies, such as 5G medical treatment, cloud platforms and artificial intelligence will promote mankind to enter the era of intelligent medicine (Figure 1) (1).

With the improvement of the mobility and sensitivity of robots, medical robots and automation systems will become the right assistants for medical staff. Meanwhile, the functions of service automation, human-computer interaction and deep learning can effectively improve the work efficiency of medical staff, reduce medical costs and improve patient satisfaction. This makes future medical models possible, such as artificial intelligence, intelligent medicine, and human-computer collaboration (2, 3).

Because of its high data transmission rate, low energy consumption, and good reliability and security, 5G network technology has been widely applied in the field of medical service. This study analyzes the applications of 5G network technology in the field of medical service and summarizes the applications of evidence available types such as artificial intelligence (AI) in nursing and their performances. Combined with cloud healthcare platforms and AI, the 5G network can provide integrated medical and nursing services, including vital signs monitoring, disease diagnosis and treatment, rehabilitation nursing, and daily life nursing. Based on professional, comprehensive, efficient, and continuous nursing measures, academia and industry are striving to improve the service quality and meet the personalized needs of specific groups. 5G technology has been widely used in the world, laying a good foundation for the digital transformation of the whole industry (4).

The proposed solutions facilitate smart healthcare applications (including telemedicine, intelligent guidance, and mobile healthcare), thus improving the working efficiency and service level of medical staff. This review aims to evaluate and synthesize the literature for the development and application of 5G network technology in the field of medical services. The keywords used for searching the relevant literature included 5G network, personalized nursing, personalized care, artificial intelligence, robot, cloud platform, smart healthcare, and intelligent medicine. The search involved the databases Cumulative Index to Web of Science, PubMed, and MEDLINE. The articles on interventions and outcomes of intelligent individuality healthcare were selected, and animal studies, editorials, comments, and letters were excluded from the review. After duplicates were removed, 176 titles and abstracts were reviewed and screened for inclusion and exclusion criteria. A total of 143 articles were included in the final review. We evaluated the efficacy outcomes of applying the 5G network technology, the presence of intelligent medical care, and the type of AI robots involved in the services. The results of our research were synthesized and narratively discussed.

### Advantages of medical networks using 5G network technology

**Figure 2:**
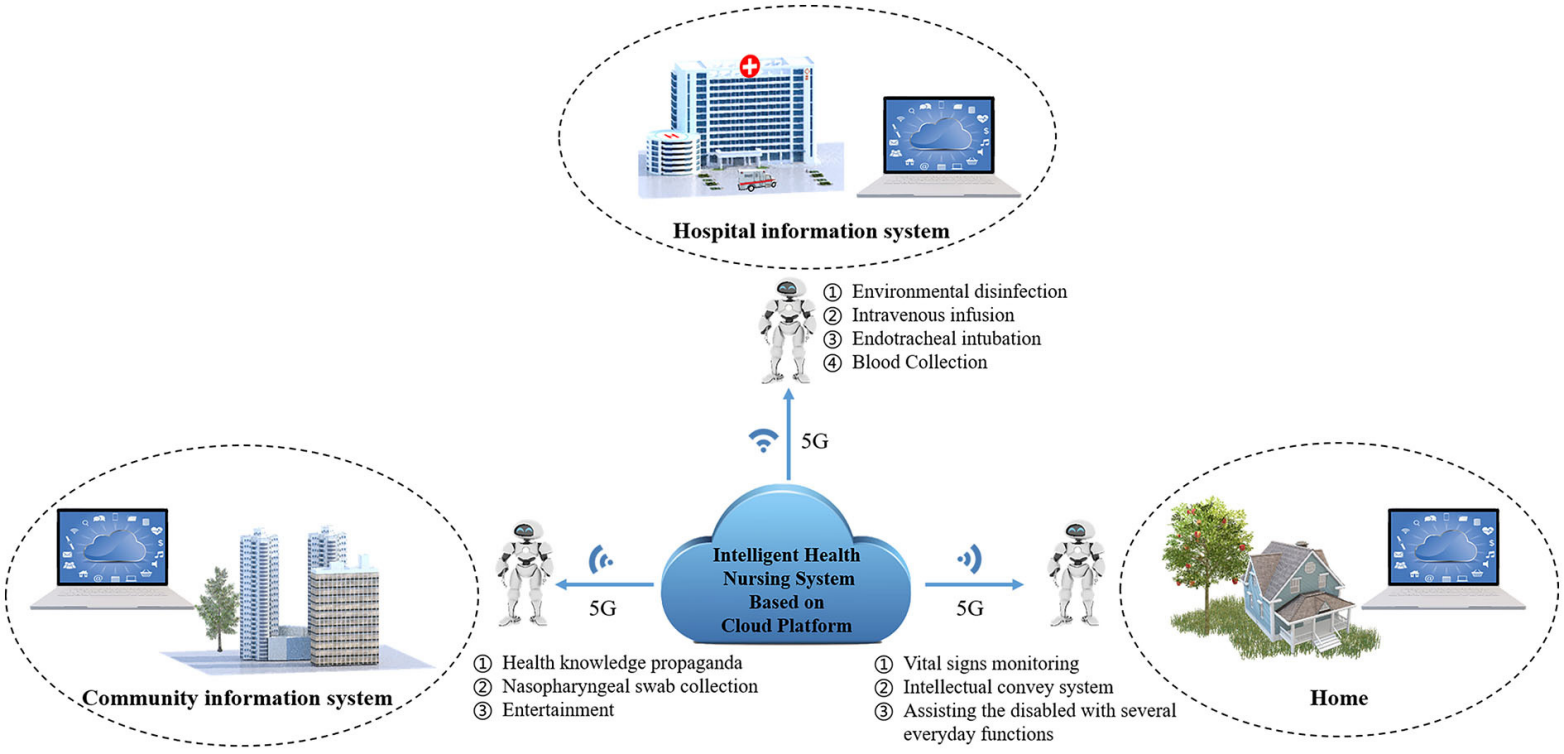
The logical architecture of 5G network combined with AI robots in personalized nursing.

With advantages such as high speed, short time, high density, and high spectrum efficiency, 5G network technology can rapidly connect people with things. Also, information transmission is free from limitations by time and space, and information utilization can be more convenient and rapid (5). First, high speed is the top advantage of 5G network technology, which allows data that occupy a large storage space (e.g., medical images) to be transmitted between hospitals in different regions or between different departments of one hospital in a short time. Based on this, the complete case data of patients can be easily accessed in regional medical treatment. Meanwhile, huge improvements in network capacity and transmission rate promote the application of technologies such as VR in the medical industry (6). Currently, the novel model of 5G-integrated cloud healthcare platform and AI robots has been gradually applied and populated in China. The model can provide standardized and personalized medical services (e.g., vital signs monitoring, drug delivery, health education, intelligent Q & A, remote visit, remote ward-round, intravenous infusion, mobile ward-round, logistics) to different patients based on the high bandwidth and low delay of 5G networks in hospitals, communities and families, fundamental functions of robots (e.g., autonomous walking, intelligent information recognition, transportation), and extraordinary computing power and professional database of cloud healthcare platforms (Figure 2).

### Applications of 5G network technology in the field of medical service

**Figure 3:**
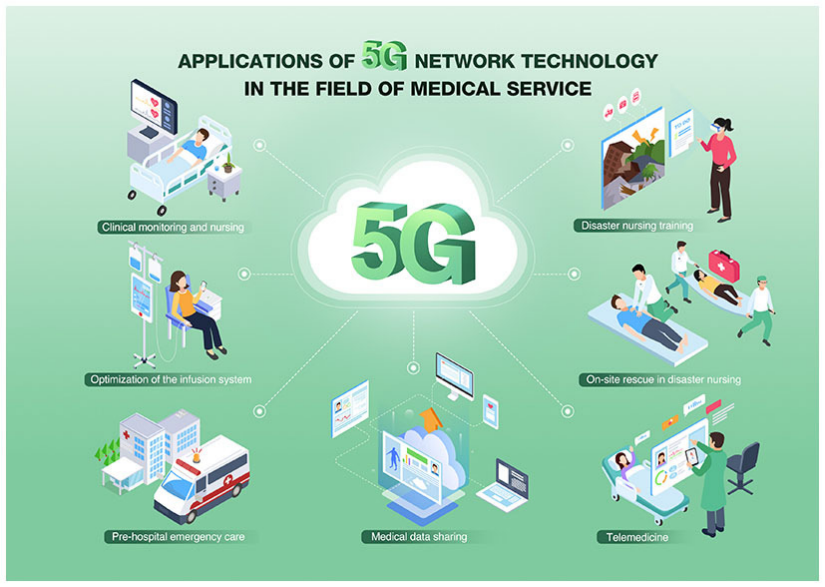
Application of 5G technologies in medical field.

With the continuous development of social informatization, medical services must seize the opportunity of 5G network technology to meet growing demands in modern medical service (7–10). The application of 5G network technology in the medical network will improve medical service quality and medical efficiency and enhance the patient experience, thus improving the service level of the entire medical industry (Figure 3) (10–15). In hospitals, 5G network technology can help to realize wireless monitoring, wireless infusion, mobile nursing, real-time patient position acquisition and monitoring, and real-time access to diagnostic images of patients (16–19). Such applications have high requirements for network isolation, security, and reliability because they are related to patient privacy. Meanwhile, image downloading and data acquisition have high requirements on bandwidth due to a large number of users and frequency utilization. With the help of THE 5G smart healthcare private network, mobile ward-round, wireless monitoring, medical image access, and mobile prescription can be achieved in hospitals. This will reduce the working intensity of medical staff, enhance service efficiency, and reduce inter-departmental coordination time, thus providing good services to patients and improving the satisfaction of medical service (20–22). Additionally, telemedicine consultation, remote examination, and video teaching can be achieved between different hospitals, attributing to significantly enhanced medical staff skills in primary medical units and medical services in remote areas. In this way, medical resources, hierarchical diagnosis and treatment, and mutual medical assistance can be integrated, and patients do not have to travel a long distance to major hospitals. For emergency rescue outside the hospital, early intervention (observation and treatment) of critical patients can be executed, and professional medical services can be provided to patients during golden rescue time to improve the cure rate.

### Clinical monitoring and nursing

Mobile nursing and wireless ward-round have been partially applied in medical service. Owing to the wide applications of 5G network technology, mobile monitoring and nursing will be popularized and become more intelligent. For instance, wards such as ICU and NICU provide more comprehensive care for patients, while the main monitoring devices are generally connected to the 5G wireless network to ensure the life safety of patients at all times. A mobile nursing system (23). A smart nursing system can be established in the ward area based on 5G network wireless technology to measure and record patient indicators such as body temperature, heart rate, and blood pressure. Meanwhile, physical sign forms and nursing evaluation forms of different patients can be issued accordingly. Additionally, the application of 5G wireless network technology in clinical nursing can help to arrange nurse work, thus improving nursing efficacy.

The first 5G hospital mobile nursing PDA was launched to accelerate the establishment of new smart hospitals based on 5G network technology (24–27). Based on this, paramedics can obtain basic and nursing information about the patient by scanning his/her wristband bar code using a 5G nurse PDA handheld terminal. Meanwhile, each patient is evaluated individually, and personalized nursing plans and health education are provided to effectively relieve the work pressure on nurses. Besides, before infusion, the medical staff uses the PDA to scan the patient barcode and infusion bag barcode to guarantee infusion safety and eliminate infusion errors.

5G network and AI are two hot topics in the field of science and technology. As a novel communication infrastructure, 5G technology provides a basis for efficient and reliable transmission of a huge amount of data and information. AI achieves information learning and evolution with a cloud brain and a neural network. Their combination will promote the development of ward-round robots that can realize real-time remote ward-round to reduce the burden on nurses (28, 29). In terms of disease prevention, diagnosis, treatment, and nursing, 5G network technology supports real-time transmission of huge data on human health and helps medical institutions in continuous physical monitoring of wearers. In this way, continuous monitoring and sensory processing devices are developed, and real-time data of patients are continuously collected. Based on these data, AI can record and analyze individual patients comprehensively and continuously so that personalized healthcare schemes can be formulated. Meanwhile, doctors can make remote judgments and analyses according to relevant data such as medical records and images, and provide personalized treatment and nursing schemes in time (28, 30, 31). Additionally, the 5G+AI system enables multi-party simultaneous consultation and connection with other hospitals and peers in a 7 × 24 manner, and multidisciplinary consultation can be held anytime, thus enhancing diagnostic accuracy.

Kuroda et al. (32) proposed a clinical sensor network system to complete data input that is previously done by nurses, thus enhancing nursing efficiency and security. Meanwhile, intelligent management of special patients can be achieved using the Internet of Things (IoT) and 5G network technology. Through portable devices, each patient can be accurately positioned and tracked so that the nursing management is humanized. The Sichuan Cancer Hospital developed a 5G medical private network consisting of remote CT imaging, AI sketching, and remote radiotherapy, which is significant to tumor treatment. Also, 5G networks can determine the specific location of a patient, which makes a great contribution to healthcare. For instance, if a patient has an emergency outside the ward, the medical staff can quickly and accurately position him/her to avoid accidents.

### Optimization of the infusion system

The application of 5G wireless network technology in hospitals can help to solve the important task of infusion system optimization (16, 33). Outpatient and emergency infusion rooms involve a series of steps, including dispensing and puncturing, and mistakes may occur in the infusion environment if there are a large number of patients. With the assistance of 5G wireless network technology, a mobile infusion information system can be developed, and paramedics can check the patient's identity and bar code of medicine to avoid mistakes. Additionally, the specific execution records of paramedics can be synchronized to the mobile infusion information system, providing immediate feedback on patient needs and enhancing nursing efficacy.

### Pre-hospital emergency care

In cases of emergency such as chest pain, first aid is of great significance. 5G network and AI-assisted equipment allow communication between the ambulance and experts in the hospital and transfer vital signs, images, and other critical information in real-time (34, 35). In this way, guidance by experts can be delivered as early as possible, and emergency nurses can access the personal information, vital signs, and examination results of the patient at any time so that the best opportunity for treatment can be obtained (36). Additionally, sufficient preparations before admission can be made to make full use of time for treatment (37).

### Disaster nursing training

Paramedics play an irreplaceable role in disaster relief. At present, only a few hospitals and medical colleges in China, offer teaching content related to disaster nursing. The incomplete knowledge system and absence of training for clinical nurses lead to an insufficient reserve of paramedics for disaster relief.

Due to the unique characteristics of disaster nursing, it is difficult for paramedics to practice in real situations. Current training methods mainly include scenario rehearsal combined with problem-based learning, hierarchical training method, action learning method, and multi-station simulated rehearsal method. The training specialized for disaster nursing needs to be optimized. The application of 5G network technology can change both training methods and concepts (25). 5G teaching enables trainers to constantly update their knowledge. Meanwhile, it makes the learning of trainees more autonomous and equips trainees with more learning choices, stronger practical capability, and more careful logical thinking. Especially, the combination of 5G network technology with virtual reality simulation and AI can simulate various disaster scenarios. It can convey information in multiple ways (e.g., text, image, sound, animation, and video) and attract the attention and embodiment of trainees (38). In this way, trainees can improve their emergency-dealing capability and operation capability by training in virtual scenarios created by real-time simulations, without limitations by time, space, and resources.

### On-site rescue in disaster nursing

Nowadays, various disasters (e.g., tsunami in the Indian Ocean, Wenchuan Earthquake, and floods in South Africa) occur frequently, and medical rescue is facing a severe situation. Doctors and nurses are exposed to challenges that are more arduous than those in any other time of history. However, disaster nursing in China started late and has a huge gap with developed countries. Therefore, advanced technologies should be actively combined to improve the personalized nursing quality of paramedics in on-site rescue, thus enhancing rescue efficiency. With the assistance of 5G network technology, rational decision and allocation of first-aid resources for medical support in large-scale disasters and emergencies can be achieved using AI to enable effective and efficient handling of various disasters or emergencies by paramedics (25). The high-tech equipment based on 5G network technology (e.g., 5G ambulances, high-resolution remote video interactive systems, VR real-time panoramic experiencing systems, GPS positioning systems, UAV, and rescue robots) can provide real-time, accurate, and efficient recording of vital signs and pathophysiological information of patients (13, 39–41).

The development of the equipment also integrates cloud transmission and analysis technology, medical examination, and data transmission techniques. Additionally, remote consultation and operation can obtain comprehensive yet real-time information about patients, and even provide guidance and remote operations in specialized scenarios such as wound treatment and intravenous infusion nursing, thus improving nursing quality and efficiency.

### Telemedicine

In the post-epidemic era, it is important to reduce the period and times of patients going to the hospital, simplify the diagnosis process, shorten the treatment period and facilitate patient treatment by using the Internet and 5G information technology (16, 42–44). Some hospitals in China have distributed electronic bracelets to patients to upload their temperature, heart rate, and blood pressure of the patients to the database as long as the electronic bracelets are with the patients every day. Then, the uploaded data are analyzed to provide guidance on diet, psychology, medicine, functional exercise, and intervention in dangerous situations. Meanwhile, the devices consist of reservation and registration, remote service, and emergency warning systems, which automatically make an appointment for the hospital process in cases of abnormal data uploading or vital signs to ensure a smooth medical process.

With the support of 5G network technology, information channels between hospitals, nursing institutions or communities have been developed, and services meeting diversified and personalized home care needs have been provided (13, 44, 45). Based on this, remote discussion on death and discharged medical records and remote teachings such as protection training and safe nursing training are conducted in an end-to-end mode and multi-party meeting. Meanwhile, intelligent data collection and process recording can be achieved by wireless communication and sensing technologies. The results are given after cloud processing and data analysis to make patient monitoring outside the hospital more convenient and efficient.

In the future, China will face severe population aging, and the incidence of senile diseases will increase correspondingly, resulting in increased demand for medical services. At present, China is exposed to an unbalanced distribution of medical resources and limited access to medical treatment in remote and under-developed areas. In this case, the growing demand for medical resources can only be satisfied by optimizing medical resource allocation and improving medical service efficiency. Patients in some areas can enjoy remote expert consultation and treatment in local hospitals or at home. Attributed to the wide application of 5G network and wearable devices and real-time transmission of vital signs and examination results, online diagnosis and treatment can be obtained regardless of regional restrictions, realizing rational allocation of medical resources (12, 46, 47). In this way, treatment efficacy is enhanced, and treatment time is reduced. Meanwhile, online teaching, nursing teaching, remote operation guidance, and case discussion can be made accessible to the medical staff at the grassroots level to improve the medical efficacy in remote areas.

### Medical data sharing

Currently, medical services in a hospital are relatively independent. Specifically, ultrasound and imaging examination, blood examination, treatment, and nursing are completed in different departments. Diagnosis and treatment require access to raw data information, including CT and MRI, which is impossible without information sharing between different departments. Currently, most hospital information systems support information sharing within the hospital. However, nurses have no immediate access to the historical data of patients kept by other medical institutions due to the limitation of network communication and transmission capacity. As a result, further development of regional medical treatment is limited. With advantages of large bandwidth, short delay, and network slicing, 5G networks can facilitate network interconnection, information sharing, and rational allocation of medical resources among different medical and health institutions in the region (15, 48–50).

### AI robots

**Figure 4:**
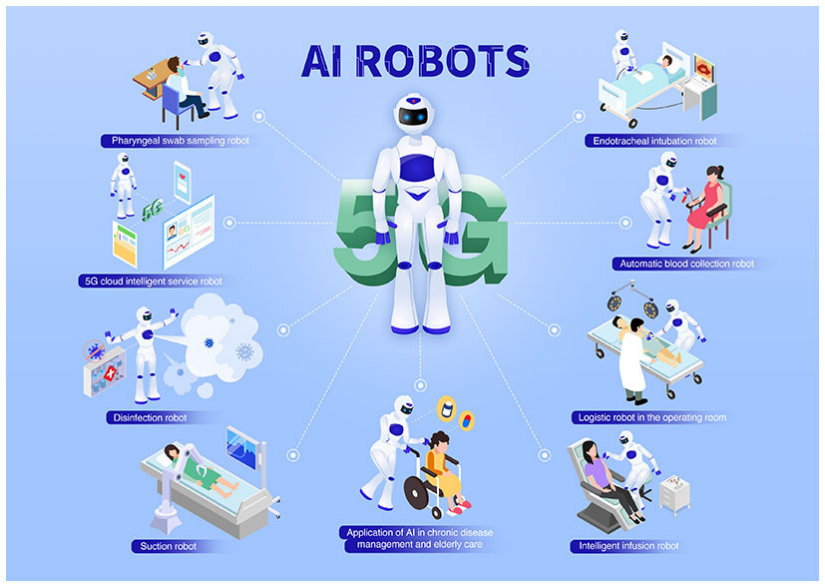
Application of AI robots in personalized nursing.

As an emerging discipline, AI is developed based on computer science, neuropsychology, philosophy, linguistics, control, and information theory (51). AI has been in the field of nursing for over four decades. Medline database was first mentioned in 1985 when expert systems were introduced to provide clinical decision support (52). Joseph Engelberger, the “Father of robots”, invented the intelligent nursing robot “Helpmate”. It is mainly used to provide smart care to the elderly living alone, and it can independently complete nursing work such as medicine delivery, meal delivery, nursing, and accompanying (53). The research on AI in medicine and health has grown rapidly in the last decade (Figure 4) (54).

### Pharyngeal swab sampling robot

Under the background of the COVID-19 epidemic, rational use of pharyngeal swab robots can prevent the infection risk of medical staff exposed to nucleic acid testing, standardize nucleic acid sampling by pharyngeal swab, and improve the quality of oral nucleic acid sampling by pharyngeal swab. The robot system can realize the nucleic acid sampling and the examination of human oral respiratory tract pharyngeal swabs under visual guidance. It mainly performs initial positioning of the human intraoral cavity, taking throat swabs, oral throat swab nucleic acid sampling, and storing throat swabs. During the COVID-19 epidemic, the first pharyngeal swab sampling robot in China was proposed by a team led by Zhong Nanshan. The proposed robot can realize high-quality pharyngeal swab sampling and cause no adverse reactions to the subjects (55). It can be used for automated nucleic acid sampling of subjects by hospitals or communities. In the near future, personalized localization of individualized oral cavity, automatic extraction of the pharyngeal swab, automatic nucleic acid oral sampling, packaging and storage of pharyngeal swab after sampling, and recording of the information of the testing subject may be achieved. Combined with 5G networks, network data sharing with different regions will be enabled, which is of great significance to the prevention and control of the epidemic.

### 5G cloud intelligent service robot

5G cloud intelligent service robots can guide patients and broadcast epidemic prevention knowledge, thus greatly reducing staff workload while meeting the personalized needs of patients. Meanwhile, the risk of cross-infection is reduced.

The outpatient department of the first affiliated hospital of USTC (Anhui Provincial Hospital) imports AI technology to assist patients in APP registering, payment, triage, and report printing. It supports multiple interacting modes (e.g., sound and image) and can provide information broadcasting, communication, and other services in non-crowded waiting areas to provide healthcare education to physical examiners, thus improving medical experience and service quality. By combining 5G networks and online platforms, this robot employs online cloud processing instead of local deployment computing, making it intelligent and highly sensitive.

### Disinfection robot

Because of their long history and mature technology, disinfection robots have distinct application advantages in the post-epidemic era (56). 5G cloud intelligent robots can realize medicine distribution in epidemic areas, preparation of disinfection solution, and floor cleaning and disinfection. For example, a robot with a disinfection water tank can complete infection following a specified route in an unmanned way. It saves human capital, improves cleaning efficiency, and greatly reduces the risk of cross-infection caused by long-term exposure to the inpatient area.

### Suction robot

Remotely controlled or automated robots can replace paramedics to effectively reduce the close contact between paramedics and infected patients, as well as the exposure to high concentration droplets and aerosols in the air, thus reducing the infection risk and relieving the psychological burden and workload of paramedics. Meanwhile, the probability of iatrogenic cross-infection can be reduced. This is especially important during the COVID-19 epidemic. Tan et al. (57) developed an intelligent suction robot, which simulates practical sputum suction by imitating the rotation of the mechanical arm and hand. The proposed robot can effectively suck out sputum in the simulation model. Furthermore, the researchers improved the mechanical arm, i.e., the motion unit of the suction robot, so that the mechanical arm is stable during sputum suction, further increasing the success rate of tube delivery. Lokomat can assist patients with stroke, brain trauma, and spinal cord injury in gait training and improve functional recovery efficiency of lower limbs, while KNRC can complete feeding and care of patients with spinal cord injury (58, 59).

### Endotracheal intubation robot

Wang et al. (60) proposed a minimized and portable remote robot-assisted intubation system (RRAIS). Animal tests revealed that the proposed robot system improved the at-first-attempt success rate and overall success rate compared with artificial laryngoscope intubation. Endotracheal intubation is performed by using the full magnetic navigation technology without opening the airway. Under guidance by an external magnet, the response magnet in the body moves to a preset target area. Meanwhile, a pilot strip is developed by placing a magnet at the tip of the endotracheal intubation guide. The tip can flexibly change orientation under a magnetic field. Additionally, the tip of the pilot strip can be shifted to the trachea by loading the external navigation magnet in the anterior cervical area. The feasibility and relatively high success rate of endotracheal intubation robot systems have been verified by human models and/or animal tests. However, these robots remain semi-automated and require assistance from paramedics (insert the machine and the endotracheal tube into the mouth). Thus, the unmanned, intelligent and personalized functions of these robots require further optimization.

### Automatic blood collection robot

Venipuncture is the most common clinical surgery globally, with 1.4 billion performed each year in the USA alone. However, 27% of patients are exposed to unclear veins, 40% of patients are exposed to invisible veins, and 60% of patients are too thin for venipuncture. Leipheimer et al. proposed a blood collection robot (VeniBot), which is composed of an ultrasonic-image guided robot (drawing blood from veins) and integrated equipment consisting of a sample processing module and a centrifuge-based blood analyzer (61). Balter et al. (62) proposed a venipuncture robot that adopts near-infrared and ultrasonic imaging technology to select injection sites and insert the needle into the blood vessel center using a 9-DOF robot under image and force guidance. Also, a medical device is developed for end-to-end blood detection and providing diagnostic results at the nursing site fully automatically.

The Magic Nurse company developed a blood collection robot that can automatically achieve full-chain blood sample collection, including loading of blood collection vessel and needle, binding pulse-pressing belt, identifying venous vessels, spraying disinfection solution, accurate puncture, control of blood collection volume, and blood sample mixing. During the operation, the robot detects the vascular conditions of the patient to intelligently determine the personalized position, orientation, and angle of puncturing. The patient is only exposed to slight pain when puncturing and no pain after puncturing.

By combining machine vision technology and intelligent navigation control technology based on biometric technology and biometric-based intelligent navigation control technology, the blood collection robot can accurately identify the position, depth, and direction of blood vessels. Also, it can intelligently plan and navigate the puncturing path. In practical applications, the robot achieves a high success rate in blood collection and can realize intellectualization, informatization, and standardization of vein blood collection.

### Logistic robot

The high-value consumables used in the operating rooms are characterized by a huge amount, various classifications, and high prices. Thus, researchers from the Bio-simulation Research Center in Nagoya, Japan developed the robot RI-MAN by combining organic materials with intelligent sensors. RI-MAN can safely transport patients and complete transfer operations, which is previously done by nurses (63).

The unique automatic delivery robot automatic transmission system takes the mobile robot as the carrier, and it is an autonomous driverless automatic handling system powered by batteries. With the popularization and promotion of various advanced sensor technologies and information technologies (e.g., positioning, obstacle avoidance, identity recognition, and automatic charging), the new intelligent logistics solutions can greatly improve the efficiency of logistics distribution in the operating room and save a lot of manpower (2, 3).

Shanghai Ruijin Hospital developed a logistic robot called NuoYa based on 5G, AI, and unmanned driving technologies. This robot can realize “dynamic object recognition”, “intelligent scheduling”, and “intelligent IoT” in the hospital and create a perfect full-scene, intelligent and real-time scheduling system for hospitals, achieving automatic distribution of materials within the hospital. 5G networks with ultra-large bandwidths are employed to transmit high-definition images around the robot to the server in real-time. Meanwhile, obstacles are identified and tracked through the deep learning algorithm. In this way, the robot can move freely, safely, and efficiently in the complex environment of the hospital. In Renji Hospital affiliated with Shanghai Jiaotong University, logistic robots have been used in operating rooms for the transportation of high-value consumables, instrument packs, and quilts. With these robots, utility nurses that walk frequently in and out of the operating room are not needed, and whole-process closed-loop management of materials can be achieved in the operating room. Additionally, human errors can be prevented, transportation efficiency can be improved, and human capital can be saved. For example, two robots can cover 20-25 operating rooms, and each robot can save about 30 min of round trips by nurses each time. The nurse may use this robot to carry medications, food, drinks, blankets, newspapers, and other important supplies to patients. The robot is equipped with infrared sensors for line-following and obstacle identification (31).

### Intelligent infusion robot

Currently, nurse shortage, low puncturing efficiency, and the unbalanced distribution of medical resources are severe issues in the medical field. The intelligent infusion robot “FUXI” developed by the Fuxi Jiuzhen Intelligent Technology (Beijing) Co., Ltd combines AI, 5G networks, and big data, and it integrates infrared image recognition, ultrasound, and pressure sensing. Based on this, its puncture success rate reaches 96%, which drastically reduces expenditure on consumables, enhances utilization efficiency of resources, and relieves mismatch of nurse resources. In the future, intelligent infusion robots will be applied in various scenarios, including home nursing and emergency support. To date, the FUXI robot has been used in over 5,000 cases of animal tests, collecting 10,000 ultrasound images of human hand veins and over 100 cases of human-based experiments. A new generation of the robots with a puncture success rate of 100% is also being developed.

### Application of AI in chronic disease management and elderly care

AI can simulate medical staff to provide individualized diagnosis, treatment, and nursing while meeting the needs of patients with chronic diseases for nursing services. Meanwhile, combined with digital therapeutics, AI can realize intelligent nursing management and clinical decision optimization. Berman et al. (64) proposed the digital therapeutics called Fare well, which consists of applications, remote sensors, and healthcare guidance provided by digital measures. After 12 weeks of diet and exercise intervention, 57% of patients exhibited decreased glycosylated hemoglobin and reduced use of medicine for diabetes. Attributed to the advances in AI, diabetes can be diagnosed, and diabetes-related complications can be predicted based on a computer-aided diagnosis (CADx) algorithm (65, 66). The CADx-based 5G intelligent diabetes monitoring systems can analyze the information of patients using big data and different machine learning measures, thus guiding personalized nursing of these patients (67).

Zeevi et al. (68) monitored the blood glucose of 800 patients for 1 week. Then, a machine learning algorithm integrating blood parameters, eating habits, anthropometric indicators, physical activity, and intestinal microbiota was proposed for personalized prediction of postprandial blood glucose. The experimental results demonstrated that AI-based personalized diet recommendations can effectively improve postprandial blood glucose and metabolism, thus providing objective references for long-term blood glucose control. Maeta et al. (69) analyzed the oral glucose tolerance test index of diabetic patients using the XGBoost machine learning algorithm. The method can effectively identify early symptoms and impaired glucose metabolism of Type-II diabetes (T2DM). Also, it helps to analyze disease progression and risk trends of patients, thus facilitating accurate treatment and nursing. These personalized AI-based interventions can effectively prevent and treat diabetes, improve the daily management of diabetes care, and reduce the risk of long-term complications (70).

In the medical field, the most mature application of AI is in intelligent elderly care (Table 1). By 2020, empty nesters in China would reach 118 million. Therefore, providing personalized nursing to the elderly, especially empty nesters, is of great significance. In the 1990s, developed countries such as USA and UK started the research on accompanying robots. Early robots were anthropomorphic ones with simple structures, stiff movement, and limited practicability.

**Table 1:** Different types of home nursing robots.

**AI robots in home nursing**	**Functions and applications**
Accompanying robot	With the shape of a cute seal, the Paro (71) robot makes a vivid response (e.g., excited, sad) through the limbs to tactile stimulation of the machine (51). It can also adjust the patient's mood and monitor psychological changes.
	Elli.Q robot (72, 73) can collect information through communication with the elderly, comprehensively analyze his/her preferences and habits, recommend personalized activities, and monitor physical condition.
Intelligent diet robot	Handy 1 robot (72, 73) uses a five-DOF (degree of freedom) mechanical arm and three detachable trays to meet different needs. The laser scanning system takes food, while the mechanical arm puts the food into the patient's mouth.
	Winsford Self-Feeder robot (74) assists in eating using two mechanical arms. One mechanical arm is equipped with a spoon, while the other pushes the food on the plate into the spoon. When eating, the patient touches the jaw switch and the mechanical arm assists the patient to eat.
	My Spoon robot (75, 76) comprises one six-DOF mechanical arm fixed to the plate bottom and one fixed bowl. The user can operate by jaw movement, foot movement, or manual operation. It can assist patients paralyzed below the neck and elderly with stiff limbs to eat by themselves.
Logistic robot	Logistic robot NAO (77) can recognize images and sounds and perceive the surrounding environment through a CPU installed in the brain, a touch sensor, and a sonar system. It can complete the operation by sound and image recognition to realize a fully programmed process.
	Herb robot (78) can accurately identify the surrounding objects or environment by using sensors and non-visual signal devices to handle and transfer the elderly.
	Robear robot (79) can lift the elderly with a low walking ability off the bed and assist user to move. The machine is equipped with sensors, allowing a high-precision tactile perception of the robot. It can obtain the body mass index of the user to be moved upon touching him/her.
Nursing robot for disabled elderly	PerMMA robot (80): patients can operate the robot through a variety of interactive interfaces (e.g., microphone, joystick, or screen touch) according to their personal needs. The robot can handle daily affairs, including cooking, dressing, and shopping.
	Walking-assistant robot Welwalk WW-1000 (81) is specially designed for stroke patients and other patients with limited movement on one leg. A bracket with a motor is fixed between the knee and the lower leg to assist the user in flexion and extension. Also, it can help the user to keep still and maintain balance during movement.
	Life-assistant robot Human Support Robot (HSR) (82) can be operated remotely through voice and/or tablets to pick up objects on the floor or shelves far away.
	Nursing robot Robear (83) is equipped with an intelligent rubber, touch sensor, and torque sensor. The robot can sense the user and prevent him/her from injury. It can carry the elderly and provide support for his/her standing and walking.
	VGo robot (83) can realize telemedicine (disease monitoring and consultation communication) and promote personalized medical service between doctors and patients and health management. It can also monitor patient recovery and answer questions about health and medicine at home.
Chronic disease nursing robot	Kompa robot (84) can monitor the vital signs of the elderly in real-time and deliver the latest symptoms to the doctor via email. It can generate shopping lists and establish video conferences to facilitate doctor consultations.

By utilizing the AR technology, the operator can determine whether the remote robot follows the intended motion of the operator or fails due to overload or some problems. Meanwhile, the use of AR and VR technologies can facilitate remote control without depending on complicated platforms. The recent development of AR and VR technologies is creating new crossroads for which we are just beginning to understand (Table 2) (91).

**Table 2:** Smart healthcare with 5G+VR/AR.

**5G+VR/AR**	**Applications**
Human system interface (intelligent robot)	It enables users to send and receive sensations in real and virtual environments (85).
VR-based Microsoft Kinect REMOVIEM system	It can be used for various home-based physical rehabilitation therapies especially for older adults (86).
Robot with VR telepresence	It can significantly reduce the operator's cognitive workload (87–89).
Remote-controlled robots with VR	It can complete common nursing duties inside hazardous clinical areas, thus helping to reduce the exposure of healthcare workers to contagions and other biohazards (89).
Robot-assisted optical camera communication (OCC) System	It can monitor the health conditions of people at home or in a hospital (90).

Medical cloud VR/AR can render and model real-time computer images. Based on the large bandwidth and low delay of the 5G technology, the security and accuracy of telemedicine and nursing can be greatly improved, and AI can provide personalized medical and nursing solutions (4). Also, the VR technology coupled with the 5G network is featured with high resolution, high fluency and high authenticity, which makes VR scenes and sensory stimulation more realistic and refined, This will greatly improve the effectiveness of rehabilitation treatment and reduce the occurrence of VR stickness (1).

An Internet of an entirely new dimension will be created for machine-machine and human-machine interactions, which can provide a supper coverage network with low latency, high reliability, and high security. These are the changes for constructing real-time interactive systems (92). The combination of AI units, IoT devices and 5G communication services can transform the traditional healthcare scenario into a new scenario (Table 3) (94, 111–115). AI and 5G communication can assist clinicians and nurses by providing remote care settings directly to the patients (116). Meanwhile, the advanced analytics in AI machines can help decision-making and disease diagnosis, and the remote monitoring of patients helps to reduce hospital stays and prevents re-admissions (94, 95).

**Table 3:** Smart healthcare with 5G+artificial intelligence.

**5G+artificial intelligence**	**Applications**
Artificial humanoid robot	It can activate memories and emotions and can be accompanied by training programs that help people to accept nonhuman relationships (93).
The advanced analytics in AI robot	It can make decisions and disease diagnoses effectively (94, 95).
Remote monitoring robot	It helps to reduce the length of hospital stay and prevent re-admissions (94, 95).
Rehabilitation robot	It can sense the human kinematics and physiology data of patients through various sensors, and formulate a reasonable treatment plan (96).
Remote-controlled medical robot	It keeps a track of the health conditions of patients, makes necessary arrangements for regular check-ups, and even books appointments (97).
Telepresence robot	It helps caregivers in this task by providing audio and visual feedback to the caregiver (98).
Sam robot	It assists the medical staff in providing frequent check-ups and nursing patients personally at their residents (30).
Digi robot	It is used in reminding staff to provide treatment or medicine to patients and can help enforce social distancing and ensure the safety (28, 97).
Telecontrolled robot	It can efficiently address cognitive decline issues by reminding care-receivers when to eat, drink or take medication, and do exercise (97, 99).
Guide robot	It can detect the surrounding environment and process and feedback information to help users effectively avoid obstacles (100).
Self-governing robot	It helps the nurse to interact and take care of the patients and can also always perform accurate surgeries (97, 101).
Social robot	It can just interact with humans by abiding by a set of rules and social behaviors (97, 102, 103).
Endoscopy robot	It can be used to take biopsies from the tissue to test for diseases and conditions (including anemia, bleeding, inflammation, diarrhea, or cancers of the digestive system) (97, 104–106).
Sister robot	It can help frontline health professionals communicate with patients in the isolation room and deliver essential foods and medicines as well (107).
5G-enabled Telesurgery robot	It facilitates the outreach of the underserved population as well as the much-needed collaboration among the surgeons across various centers in real-time (25, 108–110).
Tele-nursing robot	It can gather vital signs, and perform a wide range of manipulation tasks in a quarantine area (89).

The interactions between patients and doctors have become easier and more efficient, which leads to higher service satisfaction. If any disorder or emergency occurs when monitoring patients through data analysis, an alarm will trigger the smart emergency services (e.g., ambulance) automatically with the patient's details such as health reports, exact location, possible necessary medications, etc. Though the ambulance will send the patient to a hospital, the nearest health units will also be notified about the emergency case so that the patient can obtain timely care (117). It should be noted that self-determination medicine based on the algorithm and high-speed interactive information is different from personal or individualized medicine. Especially, the diagnosis and treatment plan will be timely, dynamic, and interactive, which enables individual status feedback about lifestyle elements, behavioral factors, and treatment effects and helps patients to obtain clinical services easily (118).

With cloud computing, sufficient resources are provided to robots to help them complete computation-intensive tasks, such as emotional recognition and feedback. This extensively improves robot intelligence and user experience (Table 4) (119). Dyumin et al. (120) proposed a structure for Cloud Robot, while in Ma et al. (121) developed a household healthcare robot. In a word, the robot integrated with 5G and cloud computing has gradually become a hotspot in this field.

**Table 4:** Smart healthcare with 5G+cloud intelligence.

**5G+cloud intelligence**	**Applications**
Cloud computing robot	Sufficient resources are provided to the robot for complete computation-intensive tasks (119).
Cloud-enabled Robot	It proposes a household healthcare robot integrated with a motion sensor and camera (120, 121).
Robot with 5G cognitive system	It is used for healthcare with a resource cognition engine and a data cognition engine. It can realize cognition of resources and realize cognition of healthcare business (10).
Cloud-assisted Robot	It can communicate with people and placate them in real-time as well as detect and transfer their emotions swiftly (122–124).
EPIC-Robot	The intelligent terminal receives the emotional results and guides the robot to achieve a real-time emotional interaction between the robot and the user (10, 122).
Cloud robot	It is connected to cloud computing infrastructure and shares training and labeling data for robot learning (125, 126).
Fog robot	It is addressing technology that is based on robot systems that use fog computing for processing data and services (127).
Cloud-enabled logistic robot	The distribution of high-value consumables in the hospital by the robot can effectively shorten the time of consumables application, and the distribution and the information are accurate with cloud computing (1, 128).

The “RIBA” nursing robot is specially designed for the elderly who are inconvenient to move. The robot can steadily and smoothly lift the patient off the bed and send him/her to the toilet, bathroom, or dining room (129). The LIECTROUX nursing robot (LIECTROUX ROBOTICS GmbH.) can independently perform health status testing, medicine taking, feeding, quilt folding, and transportation to the bathroom while recording the relevant information of the patient. Besides, this robot can protect the patient from injuries and work in a 7 × 24 way because it supports wireless charging once the battery is low.

A robot for patients with Alzheimer's disease is also reported. It enables patients to actively participate in the treatment by psychological induction, psychological conversation, and psychological hypnosis. Also, it plays the music that the patient is interested in to cheer him/her up. Besides, it helps patients to do moderate exercise as physical exercise can improve physiological health and the ataxia, which is related to the cerebellum. This robot can provide personalized care for patients at different stages according to specific conditions of the patient. In this way, the physiological and psychological pressures of patients with Alzheimer's disease can be greatly alleviated.

The “Zora” nursing robot can independently perform exercise facilitating, book reading, storytelling, and communicate with the elderly through voice recognition (130). Nursing robot Care-Obot 3 can do housework and communicate emotions by language. It has been used to take care of patients with walking difficulties and empty nesters (131). Indeed, it can help the elderly with disabilities to get rid of negative feelings, such as self-abasement and feeling lost (they regard themselves as a burden to the family and the society).

## Challenges and prospects

Despite the wide application of hospital information systems, it is still challenging to integrate 5G networks with hospital information systems. First, cyber security should be further improved: applications of 5G network technology in smart healthcare involve multiple parties and aspects, which are exposed to a high security risk. Meanwhile, the application of 5G networks allows data sharing in the medical industry, but there are differences in the informatization degree of medical institutions in different regions, bringing risks to the quality and information security of medical data. Therefore, it is crucial to further strengthen the security of 5G networks. Besides, AI systems collect data such as vital signs, health conditions, eating habits, and medication details. Indeed, should actively participate in the design and optimization of AI systems. Also, AI experts should work with ethicists to develop corresponding systems and norms, establish an early warning system, clarify the responsible party for AI-induced risks, and standardize the authority of data collection and application.

Due to its late start, the smart healthcare industry in China suffers from a severe contradiction, and the supply-demand connection is ineffective, resulting in a dilemma where the platform is present, but the service is not. Meanwhile, smart healthcare is currently limited in the research in hospitals and colleges, and thorough market analysis is lacking. As a result, products/services provided cannot meet the diverse, multi-level, and personalized medical and nursing needs of the patients.

On the other hand, the effective need for smart healthcare is limited. Due to influences by traditional concepts, living habits, and education level, patients exhibit extremely limited cognition and acceptance of smart healthcare, and they have a low capacity for using smart products and insufficient financial capacity for smart healthcare services, resulting in a significant reduction of the effective need of smart healthcare by patients with chronic diseases. Besides, the application of AI in nursing leads to a novel nurse-patient relationship, as well as new challenges in nursing (132). Parviainen and Pirhonen (133) claimed that AI is an intermediate between nurses and patients and it allows reduced contact between patients and nurses at the cost of humanistic care. Sparrow and Sparrow (134) believed that AI cannot provide emotional communications, so patients tend to feel no respect.

Currently, AI nursing robots can perform reading and storytelling but cannot meet the emotional needs of patients. Meanwhile, this communication mode reduces communication efficiency and may intensify contradictions. AI nursing supports a wide range of functions, but it is limited by low applicability and feasibility. Due to the absence of paramedics in the early stage of design and development, most AI products cannot predict and solve practical problems. Besides, AI nursing leads to a situation where personalized nursing is not possible because patients and nurses can only passively accept and use existing functions. However, nurses are often not involved in the early analysis, development, and design phases of precision medicine and AI, and they only contribute their expertise in the late phases of testing (135). Moreover, nurses' involvement in AI research and co-design is also constrained by the lack of a common vocabulary and understanding between the experts in nursing and technological domains (136).

Social interaction is a huge challenge for robotics because of its significant perceptual demands. When a response to a social cue or body language is delayed, it may be interpreted as uncertainty or mistrust. Thus, it is necessary for robots in the workplace to better understand people and their intentions as well as predict their movement. Meanwhile, considering that safety is vital, robots must know what is in the environment and take action correspondingly. To achieve this, one approach is to study body language from body movements, postures, and facial expressions.

Another major limitation in applying these wireless technologies in health systems is inaccessibility to human emotions. The technology-based health interventions (including 5G) are not widely used in this field due to the lack of proximity of these modern modalities to the major stakeholder, i.e., the patients. Recently, researchers are trying to overcome this limitation by introducing the 5G-based cognitive system (5G-Csys) with speech emotion recognition (10).

Due to some problems in their development and application, most robots can hardly be practically applied. Thus, user-friendly robots with high security, low cost, and high flexibility are urgently needed to mitigate the present challenges and provide intelligent and user-friendly nursing services.

More attention should be paid to cyber security management because the medical industry has high requirements for security, reliability, and quality of data. To guarantee medical safety and protect patient privacy, we should propose a novel safety management mode, and intensify research and development in cyber security. Meanwhile, we need to improve safety management rules, operating procedures, and technical specifications to ensure good safety and reliability of the 5G smart medical network, data, and equipment. In 2017, AI experts in the USA jointly signed the Asilomar AI Principles (The 2017 Asilomar conference) and appealed to AI professionals globally to follow this principle to guarantee the future interest and safety of human beings. Besides, issues in security and privacy induced by data acquisition and application were emphasized.

The medical private networks and a balanced development should be promoted. At present, the levels of medical information networks used by medical institutions in different regions are drastically different from each other. However, an absence of network conditions may lead to the unavailability of some equipment or software systems, thus hindering the development of smart healthcare. Therefore, it is urgent to accelerate the construction of medical private networks, especially the fundamental networks in under-developed areas. In this way, access to the large 5G network can be provided, thus laying a solid foundation for the development of telemedicine.

Tactile Internet introduces a new dimension to human-computer interaction through building a real-time interactive system with low latency. 5G network plays an important role in the wireless field and shows its breakthrough potential (137), which greatly enhances the ability of touch and skill transmission (138), and realizes immersive remote operation and interaction with the physical world.

At present, only scholars adopt 5G technology and the latest development of AI and robotics to propose the new concept of 5G tactile Internet (139), but the combination of the three is less applied to the field of intelligent medicine. Remote diagnosis, remote surgery and remote care are components of many potential applications in the tactile Internet, and they can remotely provide real-time control and physical tactile experience (138). The popularity of the 5G network will provide an opportunity for remote implementation of personalized diagnosis and treatment and nursing.

The significance of setting a clear social and legal boundary for the relationship between robots and humans should be emphasized to guarantee the safety and privacy of the caregivers and patients. In future developments, more multipurpose robots should be developed to assist caregivers in various situations, and this can be realized through more cooperation between developers and caregivers (31).

In the future, computers will play a more complementary role in clinical applications by automating the routine matters that medical staff must conduct. In this approach, more space will be created for computers to participate in non-routine health decisions that humans can manage better. Meanwhile, doctors and nurses will likely promote the use of information technology because of its benefits. They can exploit their intuition and experience to oversee the working of algorithms and AI. Moreover, medical workers can manage personalized care better than a machine (116).

The original intention of AI nursing is to provide improved personalized nursing service and alleviate the shortage of paramedics. Hence, nurses shall actively participate in research and application of AI nursing so that AI nursing products with high professionalism, rational applicability, and excellent feasibility can be developed. Meanwhile, they should keep a detailed record of problems encountered during the use of these products to facilitate optimization. Based on 5G mobile communication technology and AI platforms, AI nursing meets personalized healthcare and nursing need, changes the current mode of medical service, and improves nursing quality. In the future, the construction of 5G intelligent hospitals will further combine the technical advancements in multiple disciplines such as biomedicine, robots, communications, and medical information to realize a real interconnection between healthcare and information technology, thus providing optimized conditions for diagnosis and treatment and nursing. The integration of computer and network communication technology into the field of medical service greatly enriches the connotation and capacity of medical information, and it allows full-path, accurate, and personalized services during the entire medical process. With the support of national policies and the premise of ensuring the security of medical information, 5G network technology will further facilitate medical transformation and improve the level of medical services so that patients can enjoy safe, convenient, and high-quality medical services.

AI robots that can provide senior care are crucial to China's aging society. To satisfy the needs of today's senior citizens, technology companies should solve this social issue by working with hospitals, medical staff and engineers, and people from different social, cultural, and humanitarian backgrounds (e.g., social scientists and public policy experts). It is important to engage more talents in the rising intelligent senior care industry to fill the technology gap and develop more user-friendly products and services for senior citizens. Besides, it should be noted that robots cannot replace humans for physical and emotional companionship.

It has been shown that personal assistant robots with support functions can assist older people to obtain better life quality through physical and mental exercises (140, 141). An advantage of telepresence robots is the timely treatment of patients with urgent needs, thus leading to shorter hospital stays (142). Meanwhile, these robots enable the user to communicate with friends and families, especially if the user is in social isolation. Besides, many of these robots are multi-functional, and they can check temperatures, analyze coughs, monitor emotions and conduct questionnaires through various sensors they are equipped with.

Machine learning is another field that should be concerned because robots need to make decisions, perform sensing, adapt to environments and learn from actions to achieve further improvements. In this approach, robots can perform more complicated tasks at a higher rate and use low-cost sensor alternatives in the future. For example, in a medical emergency, intelligent robotic systems can help an emergency medical technician (EMT) to insert breathing tubes or intravenous lines and transport the patient to the hospital. This greatly improves the ability of an EMT to provide urgent care. However, the main challenge for this is to adapt to new environments, so it is crucial to specify the task correctly for the robot.

As the number of remote-end applications increases, the fast-growing healthcare industry requires a powerful communication network to effectively connect patients, healthcare professionals, medical equipment, etc. to achieve information sharing. As the next evolution of wireless connectivity, 5G mobile networks will promote telemedicine and transform the future of healthcare delivery (143).

The promising features of 5G networks provide a basis for new exciting services, such as 5G eHealth. 5G networks can help to formulate novel eHealth solutions and deliver eHealth services globally, especially for remote caring, mobile health services, and smart pharmaceuticals (90).

As 5G and other emerging technologies confluence, such as the Internet of Things (IoT), big data, Artificial Intelligence (AI), and Machine Learning (ML), the application of these technologies to augment human capacity and improve the effectiveness of human potential will greatly change the healthcare industry. In the near future, 5G technology will facilitate novel healthcare applications and promote the development of healthcare services by integrating patients, medical practitioners, and social workers through its enhanced Mobile Broadband (eMBB), URLLC, and ubiquitous access services. Meanwhile, 5G will help to realize resource pooling of expert human resources through high-performance and reliable telemedicine (including enhanced telemedicine using the tactile Internet with haptic feedback). Besides, personalized healthcare can be achieved through the progress in big data, sensor technologies, and AI/ML. Additionally, routine activities of humans (e.g., diagnoses) will be supported by AI and ML algorithms. Based on this, the overall healthcare system will be enhanced and benefit the global economy. These are important trends in the field of healthcare driving the 5G era transition. Moreover, smart healthcare is growing quickly, and 5G will reconstruct the healthcare system by improving the quality of medical service intelligently, balancing the distribution of medical resources between urban and rural areas, and reducing healthcare costs. We are cautiously optimistic about these trends, although there is still a long way to go to achieve smart healthcare.

## Author contributions

HL and CG contributed to data collection and analysis, manuscript design, and preparation. HL revised the manuscript. Both authors have agreed to be accountable for the content of the work.

## Conflict of interest

The authors declare that the research was conducted in the absence of any commercial or financial relationships that could be construed as a potential conflict of interest.

## Publisher's note

All claims expressed in this article are solely those of the authors and do not necessarily represent those of their affiliated organizations, or those of the publisher, the editors and the reviewers. Any product that may be evaluated in this article, or claim that may be made by its manufacturer, is not guaranteed or endorsed by the publisher.
